# 5,8-Dibromo-14,17-difluoro-2,11-dithia­[3.3]paracyclo­phane

**DOI:** 10.1107/S1600536810028904

**Published:** 2010-07-24

**Authors:** Xiaowei Hao, Di Wu

**Affiliations:** a Key Laboratory of Pesticides and Chemical Biology of the Ministry of Education, College of Chemistry, Central China Normal University, Wuhan 430079, People’s Republic of China

## Abstract

The title compound, C_16_H_12_Br_2_F_2_S_2_ [systematic name: 1^2^,1^5^-dibromo-5^2^,5^5^-difluoro-2,7-dithia-1,5(1,4)-dibenzenaocta­phane], has two approximately parallel benzene rings with a dihedral angle of 1.53 (15)° between them and with a centroid–centroid distance of 3.3066 (18) Å. In the crystal structure, mol­ecules are stacked along the *a* axis through an inter­molecular π–π inter­action with a centroid–centroid distance of 3.7803 (18) Å. Mol­ecules are also connected by a C—H⋯S inter­action, forming a chain along the *b* axis.

## Related literature

For the preparation of the title compound, see: Wang *et al.* (2006 ▶); Xu *et al.* (2008 ▶). For potential applications of intra­molecular π–π inter­actions in organic reactions, see: Korenaga *et al.* (2007 ▶).
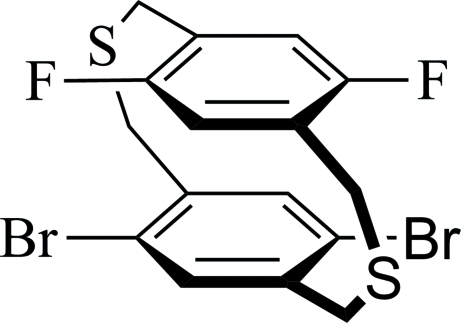


## Experimental

### 

#### Crystal data

C_16_H_12_Br_2_F_2_S_2_
*M* *_r_* = 466.20
Triclinic, 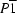

*a* = 6.9744 (5) Å
*b* = 9.6798 (7) Å
*c* = 12.9376 (9) Å
α = 72.301 (1)°
β = 75.764 (1)°
γ = 76.535 (1)°
*V* = 794.63 (10) Å^3^
*Z* = 2
Mo *K*α radiation
μ = 5.38 mm^−1^
*T* = 298 K
0.16 × 0.12 × 0.10 mm

#### Data collection

Bruker SMART APEX diffractometer
Absorption correction: multi-scan (*SADABS*; Sheldrick, 1996 ▶) *T*_min_ = 0.480, *T*_max_ = 0.616
4952 measured reflections
2920 independent reflections
2493 reflections with *I* > 2σ(*I*)
*R*_int_ = 0.030

#### Refinement

*R*[*F* ^2^ > 2σ(*F* ^2^)] = 0.036
*wR*(*F* ^2^) = 0.096
*S* = 1.02
2920 reflections
199 parameters
H-atom parameters constrained
Δρ_max_ = 0.58 e Å^−3^
Δρ_min_ = −0.85 e Å^−3^

### 

Data collection: *SMART* (Bruker, 1997 ▶); cell refinement: *SAINT* (Bruker, 1999 ▶); data reduction: *SAINT*; program(s) used to solve structure: *SHELXS97* (Sheldrick, 2008 ▶); program(s) used to refine structure: *SHELXL97* (Sheldrick, 2008 ▶); molecular graphics: *SHELXTL* (Sheldrick, 2008 ▶); software used to prepare material for publication: *SHELXTL*.

## Supplementary Material

Crystal structure: contains datablocks I, global. DOI: 10.1107/S1600536810028904/is2571sup1.cif

Structure factors: contains datablocks I. DOI: 10.1107/S1600536810028904/is2571Isup2.hkl

Additional supplementary materials:  crystallographic information; 3D view; checkCIF report

## Figures and Tables

**Table 1:** Hydrogen-bond geometry (Å, °)

*D*—H⋯*A*	*D*—H	H⋯*A*	*D*⋯*A*	*D*—H⋯*A*
C8—H8*B*⋯S2^i^	0.97	2.86	3.801 (3)	165

Symmetry code: (i) 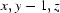.

**Fig. 1.**
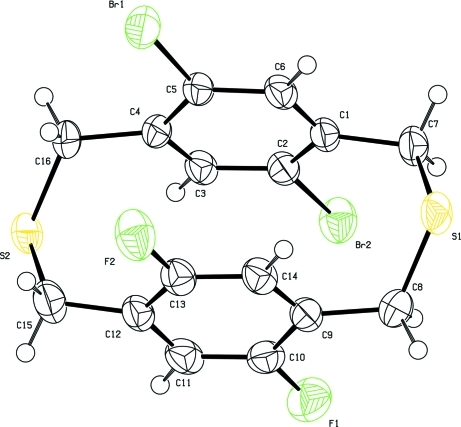
View of the molecule of (I) showing the atom-labelling scheme. Displacement ellipsoids are drawn at the 50% probability level. H atoms are represented by spheres of arbitrary radius.

## supplementary crystallographic information

### Comment

The aromatic-aromatic π–π interaction is an important phenomena in organic reactions (Korenaga *et al.*, 2007). The structural and electronic properties of these derivatives result from the characteristic interactions between the two π-electron systems. Substituents in the benzene rings can significantly effect the π–π interaction. In the compound [3,3]paracyclophane, we explored the intramolecular π–π interaction between the two benzene rings. In the crystal structure, intermolecular π–π and non-classical hydrogen bonding interactions link the molecule, in which they seem to be effective in the stabilization of the structure.

### Experimental

The title compound was prepared according to the method reported previously (Wang *et al.*, 2006; Xu *et al.*, 2008). A solution with equimolar amounts of (2,5-difluoro-1,4-phenylene)dimethanethiol and 1,4-dibromo-2,5-bis(bromomethyl)benzene in degassed THF (500 ml) was added dropwised under N_2_ over 12 h to a refluxing solution of potassium carbonate (5equiv) in EtOH (1.2*L*). After an additional 2 h at the reflux temperature 363 K, the mixture was cooled and the solvent were removed. The resulting residue was treated with CH_2_Cl_2_ (300 ml) and water (300 ml). The organic phase was separated, the aqueous extracted with CH_2_Cl_2_ three times. The combined organic layers was dried over Na_2_SO_4_, Then solvent was removed, and the resulting solid was chromatographed on silica gel using CH_2_Cl_2_/petroleum ether (1:1, *v*/*v*) as eluent. The product was further purified by recrystallization from toluene.

### Refinement

All H atoms were initially located in a difference map, and then were constrained to an idealized geometry (C—H = 0.93 or 0.97 Å). The isotropic displacement parameters were set to *U*_iso_(H) = 1.2*U*_eq_(C).

### Crystal data

**Table:**

C_16_H_12_Br_2_F_2_S_2_	*Z* = 2
*M**_r_* = 466.20	*F*(000) = 456
Triclinic, *P*1	*D*_x_ = 1.948 Mg m^−^^3^
Hall symbol: -P 1	Mo *K*α radiation, λ = 0.71073 Å
*a* = 6.9744 (5) Å	Cell parameters from 2764 reflections
*b* = 9.6798 (7) Å	θ = 2.4–28.2°
*c* = 12.9376 (9) Å	µ = 5.38 mm^−^^1^
α = 72.301 (1)°	*T* = 298 K
β = 75.764 (1)°	Block, colorless
γ = 76.535 (1)°	0.16 × 0.12 × 0.10 mm
*V* = 794.63 (10) Å^3^	

### Data collection

**Table:**

Bruker SMART APEX diffractometer	2920 independent reflections
Radiation source: fine-focus sealed tube	2493 reflections with *I* > 2σ(*I*)
graphite	*R*_int_ = 0.030
φ and ω scans	θ_max_ = 25.5°, θ_min_ = 3.1°
Absorption correction: multi-scan (*SADABS*; Sheldrick, 1996)	*h* = −8→8
*T*_min_ = 0.480, *T*_max_ = 0.616	*k* = −11→11
4952 measured reflections	*l* = −13→15

### Refinement

**Table:**

Refinement on *F*^2^	Primary atom site location: structure-invariant direct methods
Least-squares matrix: full	Secondary atom site location: difference Fourier map
*R*[*F*^2^ > 2σ(*F*^2^)] = 0.036	Hydrogen site location: inferred from neighbouring sites
*wR*(*F*^2^) = 0.096	H-atom parameters constrained
*S* = 1.02	*w* = 1/[σ^2^(*F*_o_^2^) + (0.0636*P*)^2^] where *P* = (*F*_o_^2^ + 2*F*_c_^2^)/3
2920 reflections	(Δ/σ)_max_ < 0.001
199 parameters	Δρ_max_ = 0.58 e Å^−^^3^
0 restraints	Δρ_min_ = −0.85 e Å^−^^3^

### Special details

**Table:**

Geometry. All e.s.d.'s (except the e.s.d. in the dihedral angle between two l.s. planes) are estimated using the full covariance matrix. The cell e.s.d.'s are taken into account individually in the estimation of e.s.d.'s in distances, angles and torsion angles; correlations between e.s.d.'s in cell parameters are only used when they are defined by crystal symmetry. An approximate (isotropic) treatment of cell e.s.d.'s is used for estimating e.s.d.'s involving l.s. planes.
Refinement. Refinement of *F*^2^ against ALL reflections. The weighted *R*-factor *wR* and goodness of fit *S* are based on *F*^2^, conventional *R*-factors *R* are based on *F*, with *F* set to zero for negative *F*^2^. The threshold expression of *F*^2^ > σ(*F*^2^) is used only for calculating *R*-factors(gt) *etc*. and is not relevant to the choice of reflections for refinement. *R*-factors based on *F*^2^ are statistically about twice as large as those based on *F*, and *R*- factors based on ALL data will be even larger.

### Fractional atomic coordinates and isotropic or equivalent isotropic displacement parameters (Å^2^)

**Table:**

	*x*	*y*	*z*	*U*_iso_*/*U*_eq_	
Br1	0.27806 (5)	0.76264 (4)	0.51530 (3)	0.04905 (14)	
Br2	0.44765 (5)	0.76124 (4)	−0.00392 (3)	0.04945 (14)	
C1	0.4459 (4)	0.6243 (3)	0.2275 (3)	0.0328 (6)	
C2	0.3888 (4)	0.7531 (3)	0.1493 (2)	0.0337 (6)	
C3	0.2827 (4)	0.8783 (3)	0.1795 (3)	0.0340 (6)	
H3	0.2432	0.9614	0.1252	0.041*	
C4	0.2339 (4)	0.8832 (3)	0.2883 (3)	0.0336 (6)	
C5	0.3120 (4)	0.7600 (3)	0.3655 (2)	0.0330 (6)	
C6	0.4130 (4)	0.6327 (3)	0.3353 (3)	0.0356 (6)	
H6	0.4597	0.5513	0.3887	0.043*	
C7	0.5314 (4)	0.4785 (3)	0.2002 (3)	0.0395 (7)	
H7A	0.6509	0.4346	0.2319	0.047*	
H7B	0.5709	0.4956	0.1206	0.047*	
C8	0.1518 (5)	0.4395 (3)	0.1753 (3)	0.0472 (8)	
H8A	0.2100	0.4717	0.0976	0.057*	
H8B	0.0700	0.3672	0.1829	0.057*	
C9	0.0172 (4)	0.5697 (3)	0.2108 (3)	0.0365 (7)	
C10	−0.0598 (4)	0.6907 (3)	0.1333 (3)	0.0387 (7)	
C11	−0.1606 (4)	0.8195 (3)	0.1582 (3)	0.0424 (7)	
H11	−0.2073	0.8984	0.1030	0.051*	
C12	−0.1935 (4)	0.8330 (3)	0.2650 (3)	0.0384 (7)	
C13	−0.1325 (4)	0.7082 (3)	0.3432 (3)	0.0381 (7)	
C14	−0.0278 (4)	0.5803 (3)	0.3187 (3)	0.0379 (7)	
H14	0.0134	0.5003	0.3748	0.045*	
C15	−0.2780 (4)	0.9789 (3)	0.2925 (3)	0.0480 (8)	
H15A	−0.3191	0.9615	0.3721	0.058*	
H15B	−0.3965	1.0238	0.2601	0.058*	
C16	0.0952 (4)	1.0135 (3)	0.3234 (3)	0.0425 (7)	
H16A	0.1759	1.0849	0.3192	0.051*	
H16B	0.0319	0.9801	0.4001	0.051*	
F1	−0.0293 (3)	0.6816 (2)	0.02783 (17)	0.0624 (5)	
F2	−0.1649 (3)	0.7156 (2)	0.45008 (16)	0.0599 (5)	
S1	0.35458 (12)	0.34989 (8)	0.25145 (7)	0.0429 (2)	
S2	−0.10017 (11)	1.10582 (8)	0.24325 (7)	0.0417 (2)	

### Atomic displacement parameters (Å^2^)

**Table:**

	*U*^11^	*U*^22^	*U*^33^	*U*^12^	*U*^13^	*U*^23^
Br1	0.0641 (2)	0.0407 (2)	0.0429 (2)	0.00474 (15)	−0.01841 (16)	−0.01540 (16)
Br2	0.0578 (2)	0.0461 (2)	0.0390 (2)	−0.00154 (16)	−0.00537 (15)	−0.01191 (16)
C1	0.0266 (12)	0.0259 (14)	0.0458 (17)	−0.0033 (11)	−0.0048 (12)	−0.0118 (13)
C2	0.0292 (13)	0.0319 (15)	0.0397 (16)	−0.0034 (11)	−0.0065 (12)	−0.0103 (13)
C3	0.0315 (13)	0.0252 (14)	0.0429 (17)	−0.0033 (11)	−0.0101 (12)	−0.0042 (12)
C4	0.0325 (14)	0.0243 (14)	0.0469 (18)	−0.0043 (11)	−0.0095 (12)	−0.0126 (13)
C5	0.0317 (13)	0.0273 (14)	0.0414 (16)	−0.0029 (11)	−0.0097 (12)	−0.0102 (12)
C6	0.0328 (14)	0.0272 (14)	0.0446 (17)	−0.0003 (11)	−0.0121 (13)	−0.0057 (13)
C7	0.0400 (15)	0.0286 (15)	0.0489 (19)	0.0008 (12)	−0.0068 (13)	−0.0150 (14)
C8	0.0494 (17)	0.0327 (16)	0.068 (2)	−0.0054 (13)	−0.0184 (16)	−0.0204 (16)
C9	0.0301 (13)	0.0265 (14)	0.0548 (19)	−0.0065 (11)	−0.0095 (13)	−0.0109 (14)
C10	0.0397 (15)	0.0361 (16)	0.0444 (17)	−0.0093 (12)	−0.0147 (13)	−0.0088 (14)
C11	0.0395 (15)	0.0320 (16)	0.054 (2)	−0.0047 (12)	−0.0178 (14)	−0.0022 (14)
C12	0.0303 (14)	0.0279 (15)	0.056 (2)	−0.0035 (11)	−0.0084 (13)	−0.0111 (14)
C13	0.0315 (14)	0.0377 (17)	0.0425 (18)	−0.0071 (12)	−0.0019 (12)	−0.0098 (14)
C14	0.0368 (14)	0.0278 (14)	0.0455 (18)	−0.0053 (11)	−0.0101 (13)	−0.0027 (13)
C15	0.0383 (15)	0.0348 (17)	0.066 (2)	0.0012 (13)	−0.0058 (15)	−0.0156 (16)
C16	0.0467 (16)	0.0278 (15)	0.057 (2)	0.0058 (13)	−0.0182 (15)	−0.0190 (14)
F1	0.0784 (13)	0.0609 (13)	0.0542 (13)	−0.0022 (10)	−0.0265 (11)	−0.0201 (10)
F2	0.0644 (12)	0.0585 (12)	0.0459 (12)	0.0036 (10)	−0.0028 (9)	−0.0146 (10)
S1	0.0498 (4)	0.0213 (4)	0.0546 (5)	−0.0002 (3)	−0.0115 (4)	−0.0086 (3)
S2	0.0464 (4)	0.0214 (4)	0.0543 (5)	0.0031 (3)	−0.0157 (4)	−0.0075 (3)

### Geometric parameters (Å, °)

**Table:**

Br1—C5	1.901 (3)	C8—H8B	0.9700
Br2—C2	1.904 (3)	C9—C14	1.384 (4)
C1—C6	1.381 (4)	C9—C10	1.388 (4)
C1—C2	1.391 (4)	C10—F1	1.355 (4)
C1—C7	1.510 (4)	C10—C11	1.370 (4)
C2—C3	1.377 (4)	C11—C12	1.386 (4)
C3—C4	1.377 (4)	C11—H11	0.9300
C3—H3	0.9300	C12—C13	1.376 (4)
C4—C5	1.399 (4)	C12—C15	1.511 (4)
C4—C16	1.516 (4)	C13—F2	1.367 (3)
C5—C6	1.386 (4)	C13—C14	1.367 (4)
C6—H6	0.9300	C14—H14	0.9300
C7—S1	1.823 (3)	C15—S2	1.817 (3)
C7—H7A	0.9700	C15—H15A	0.9700
C7—H7B	0.9700	C15—H15B	0.9700
C8—C9	1.507 (4)	C16—S2	1.815 (3)
C8—S1	1.822 (3)	C16—H16A	0.9700
C8—H8A	0.9700	C16—H16B	0.9700
			
C6—C1—C2	116.9 (3)	C14—C9—C8	123.2 (3)
C6—C1—C7	119.9 (3)	C10—C9—C8	120.3 (3)
C2—C1—C7	123.1 (3)	F1—C10—C11	118.9 (3)
C3—C2—C1	121.6 (3)	F1—C10—C9	118.2 (3)
C3—C2—Br2	117.3 (2)	C11—C10—C9	122.9 (3)
C1—C2—Br2	121.1 (2)	C10—C11—C12	120.4 (3)
C4—C3—C2	121.5 (3)	C10—C11—H11	119.8
C4—C3—H3	119.3	C12—C11—H11	119.8
C2—C3—H3	119.3	C13—C12—C11	116.2 (3)
C3—C4—C5	117.0 (3)	C13—C12—C15	121.9 (3)
C3—C4—C16	121.5 (3)	C11—C12—C15	121.8 (3)
C5—C4—C16	121.5 (3)	F2—C13—C14	118.0 (3)
C6—C5—C4	121.0 (3)	F2—C13—C12	118.2 (3)
C6—C5—Br1	118.4 (2)	C14—C13—C12	123.6 (3)
C4—C5—Br1	120.6 (2)	C13—C14—C9	120.2 (3)
C1—C6—C5	121.3 (3)	C13—C14—H14	119.9
C1—C6—H6	119.3	C9—C14—H14	119.9
C5—C6—H6	119.3	C12—C15—S2	113.1 (2)
C1—C7—S1	112.95 (19)	C12—C15—H15A	109.0
C1—C7—H7A	109.0	S2—C15—H15A	109.0
S1—C7—H7A	109.0	C12—C15—H15B	109.0
C1—C7—H7B	109.0	S2—C15—H15B	109.0
S1—C7—H7B	109.0	H15A—C15—H15B	107.8
H7A—C7—H7B	107.8	C4—C16—S2	115.7 (2)
C9—C8—S1	115.3 (2)	C4—C16—H16A	108.4
C9—C8—H8A	108.4	S2—C16—H16A	108.4
S1—C8—H8A	108.4	C4—C16—H16B	108.4
C9—C8—H8B	108.4	S2—C16—H16B	108.4
S1—C8—H8B	108.4	H16A—C16—H16B	107.4
H8A—C8—H8B	107.5	C8—S1—C7	103.73 (14)
C14—C9—C10	116.3 (3)	C16—S2—C15	102.73 (15)
			
C6—C1—C2—C3	−7.7 (4)	C14—C9—C10—C11	5.4 (4)
C7—C1—C2—C3	169.6 (2)	C8—C9—C10—C11	−171.2 (3)
C6—C1—C2—Br2	174.25 (19)	F1—C10—C11—C12	−179.6 (3)
C7—C1—C2—Br2	−8.5 (4)	C9—C10—C11—C12	−1.2 (4)
C1—C2—C3—C4	2.1 (4)	C10—C11—C12—C13	−4.5 (4)
Br2—C2—C3—C4	−179.7 (2)	C10—C11—C12—C15	171.4 (3)
C2—C3—C4—C5	5.5 (4)	C11—C12—C13—F2	−178.9 (3)
C2—C3—C4—C16	−172.1 (2)	C15—C12—C13—F2	5.2 (4)
C3—C4—C5—C6	−7.6 (4)	C11—C12—C13—C14	6.2 (4)
C16—C4—C5—C6	170.1 (2)	C15—C12—C13—C14	−169.7 (3)
C3—C4—C5—Br1	173.4 (2)	F2—C13—C14—C9	−177.0 (3)
C16—C4—C5—Br1	−9.0 (4)	C12—C13—C14—C9	−2.1 (4)
C2—C1—C6—C5	5.5 (4)	C10—C9—C14—C13	−3.7 (4)
C7—C1—C6—C5	−171.8 (2)	C8—C9—C14—C13	172.7 (3)
C4—C5—C6—C1	2.1 (4)	C13—C12—C15—S2	103.6 (3)
Br1—C5—C6—C1	−178.9 (2)	C11—C12—C15—S2	−72.1 (3)
C6—C1—C7—S1	70.6 (3)	C3—C4—C16—S2	32.3 (4)
C2—C1—C7—S1	−106.6 (3)	C5—C4—C16—S2	−145.2 (2)
S1—C8—C9—C14	−32.7 (4)	C9—C8—S1—C7	−73.4 (3)
S1—C8—C9—C10	143.6 (2)	C1—C7—S1—C8	64.0 (3)
C14—C9—C10—F1	−176.2 (2)	C4—C16—S2—C15	75.0 (3)
C8—C9—C10—F1	7.2 (4)	C12—C15—S2—C16	−64.9 (3)

### Hydrogen-bond geometry (Å, °)

**Table:**

*D*—H···*A*	*D*—H	H···*A*	*D*···*A*	*D*—H···*A*
C8—H8B···S2^i^	0.97	2.86	3.801 (3)	165

Symmetry codes: (i) *x*, *y*−1, *z*.
